# Cardiotoxicity of concomitant radiotherapy and trastuzumab for early breast cancer

**DOI:** 10.2478/raon-2013-0040

**Published:** 2014-04-25

**Authors:** Tanja Marinko, Jure Dolenc, Cvetka Bilban-Jakopin

**Affiliations:** 1 Department of Radiotherapy, Institute of Oncology Ljubljana, Ljubljana, Slovenia; 2 Department of Cardiology, University Medical Centre Ljubljana, Ljubljana, Slovenia; 3 Institute of Oncology Ljubljana, Ljubljana, Slovenia

**Keywords:** radiotherapy, cardiotoxicity, trastuzumab, early breast cancer

## Abstract

**Background:**

Trastuzumab therapy given in combination with one of several chemotherapy regimens is currently considered the standard of care for the treatment of early-stage, human epidermal growth factor receptor-2 (HER2) -positive breast cancer. The treatment with trastuzumab is due to a significant impact on the survival part of the standard adjuvant treatment of patients with HER2-positive breast cancer. Patients treated with postoperative breast or chest wall irradiation receive trastuzumab concomitant with radiotherapy. In a small proportion of patients trastuzumab causes cardiotoxicity. Preclinical findings indicate a radiosensibilizing effect of trastuzumab in breast cancer cells, but it is not yet clear whether it radiosensibilizes cells of healthy tissues too.

**Conclusions:**

Special attention is required when left breast or left thoracic wall is irradiated in patient receiving trastuzumab, because long-term effects of the concurrent treatment with trastuzumab and radiotherapy are not yet known. In an era where more patients are surviving a diagnosis of breast cancer, better understanding and earlier detection of therapy-induced cardiac toxicity will be of paramount importance.

## Introduction

Trastuzumab (Herceptin^®^) is a humanised monoclonal antibody that binds to the extracellular domain of the HER2 receptor, transmembrane glycoprotein and thereby inhibits cell growth and reproduction. The exact mechanism of action that leads to the clinical efficacy of trastuzumab is not yet entirely clear, although its antitumor effect *in vitro* has already been shown before 20 years. Trastuzumab was approved by the US Food and Drug Administration in September 1998 for the treatment of metastatic breast cancer. Nowadays it is widely used in the metastatic and adjuvant systemic treatment for breast cancer.

HER2-positive (HER2 over-expression of the receptor) is 15–25% of breast cancers.^1^–^3^ If untreated, they have a worse prognosis than HER2 negative tumours.^4^ Within the adjuvant treatment of patients with HER2-positive breast cancer, a therapy with trastuzumab improves the survival, which is confirmed by the four major international studies: Herceptin Adjuvant Trial (HERA), National Surgical Adjuvant Breast and Bowel Project (NSABP) B-31, North Central Cancer Treatment Group (NCCTG) N9831 and BCIRG Breast Cancer International Research Group (BCIRG) 006^th^. Among four major adjuvant trials more than 13.000 women with HER2-positive early breast cancer were enrolled.^5^ All four studies report on the extension of time to disease recurrence and the overall survival if one year of the treatment with trastuzumab is added to the standard chemotherapy (CT).^3^,^6^,^7^ As an adjuvant treatment of breast cancer in these studies patients firstly received anthracyclines and then taxanes, as monotherapy or concomitantly with trastuzumab, which was then given with total one year.^5^

The meta analysis, published in Cohrane Database in April 2012, which included eight studies involving 11.991 patients, found that the combined hazard ratios (HR) for the overall survival and disease-free survival significantly favoured the trastuzumab-containing regiments (HR 0.66, 95% confidence interval [CI] 0.57–0.77, P < 0.00001; and HR 0.60, 95% CI 0.50 to 0.71, P < 0.00001) used in the treatment for early and locally advanced breast cancer.^4^ Benefit of trastuzumab is higher if it is introduced as soon as possible in the course of the treatment and simultaneously with CT.^8^,^9^

Clinical guidelines based on these findings, therefore, recommended the introduction of trastuzumab before postoperative radiotherapy and after the treatment with anthracyclines.^10^ Because the half-life of trastuzumab is long (four weeks) and washout period is 20 weeks, it is usually administered concomitantly with radiotherapy.^11^

### Cardiotoxicity of trastuzumab and anthracyclins

The treatment with trastuzumab is well tolerated by most patients. In a small proportion of patients treatment may be necessarily temporary or permanently discontinued due to the resulting damage to the heart.^2^,^9^,^12^ In a large randomized clinical trials, where patients received trastuzumab as a part of the adjuvant treatment (after completing anthracycline CT), the reported incidence of severe heart failure and cardiac death was from 0.6% (study HERA) to 4% (NSABP-B31). The most common cardiovascular event reported was an asymptomatic decrease in left ventricular ejection fraction (LVEF). It was shown that the risk of developing heart failure was significantly higher in patients who had previously received anthracyclines.^13^ In the above mentioned Cochran’s meta-analysis published in 2012 it was reported, that trastuzumab significantly increased the risk of congestive heart failure (CHF) (risk ratio [RR] 5.11, 90% CI 3.00–8.72, P < 0.0001) and LVEF decline (RR 1.83; 90% CI 1.36 to 2.47, P = 0.0008).

Anthracyclines, which are received by most patients with early breast cancer during the adjuvant treatment with CT, may cause cardiotoxicity type I.^14^ They induce injury to myocites, probably caused by the resulting toxic oxygen free radicals. The injury is the result of oxidative stress and leads to irreversible damage of myocites (necrosis and apoptosis), which are eventually replaced by connective tissue.^15^ The rate of heart failure is dependent on the cumulative dose of medication.^14^

Trastuzumab could cause cardiotoxicity type II.^14^ The occurrence of cardiotoxicity isn’t dose-dependent. Whether asymptomatic or symptomatic heart failure, if it is caused by trastuzumab, it should be reversible. Ewer *et al*. report on the reversibility of trastuzumab-induced reduction in LVEF after discontinuation of therapy.^16^ In the heart biopsy only benign ultrastructural changes can be found.^14^

So far there is very little known about the long-term significance of asymptomatic LVEF reductions. Cardiologists warn that it is necessary to monitor these patients annually even after the treatment with trastuzumab.^15^,^17^ The mechanism of heart damage caused by trastuzumab is not yet fully understood. HER2 receptors are normally present on the myocites and they are important for the normal development and function of the heart. Preclinical studies indicate that the direct blockade of the HER2 receptor on myocites has at least partially an important impact on cardiotoxicity of trastuzumab.^18^,^19^ Among the drugs used for the treatment of patients with cancer there are some other well-known substances, that cause mild, chronic, partially reversible, but clinically silent cardiotoxic side effects, that in the opinion of the cardiologists needs a long term attention.^20^

### Cardiotoxicity of radiation therapy

Irradiation of the heart may be associated with multiple effects on the heart; the result could be acute or chronic pericarditis, pericardial effusion, constrictive pericarditis, coronary vascular disease, restrictive cardiomyopathy, valvular heart disease or malfunction of the conductive system of the heart. The development of these defects depends mainly on the dose received by the heart and the proportion of the heart, which is exposed to radiation.^14^ Another important factor for the development of heart damage, is the age. According to the literature, young people (< 20 years) have the highest risk of subsequent heart damage because the organism is still developing and cells multiply rapidly, which makes them more susceptible to damage of the DNA molecules.^21^ Heart damage due to radiation is caused by microvascular lesions, as well as direct apoptosis of damaged cells. The final outcome is fibrosis that develops over the years, after the completion of radiotherapy.^22^

Cardiotoxicity of adjuvant radiotherapy in patients with breast cancer is the subject of many studies.^23^,^24^ In the EBCTCG meta-analysis in 2005, which included 42 000 women in 78 randomised trials, it was shown that the use of radiation therapy significantly improves the disease-specific survival for patients with an early stage breast cancer.^23^ But the same analysis, comparing the trials with radiotherapy *versus* not, also reported that there was, at least with some of the older radiotherapy regimens, a significant excess mortality from heart disease (RR 1.27, standard error [SE] 0.07, 2p = 0.0001). It was slight during the first five years, but continued after year 15.

Meta-analysis, which allows the identification and abstraction of critical information from different randomized, controlled trials^25^, analysed long term mortality from heart disease after radiotherapy for an early breast cancer of about 300 000 women in United States (US) Surveillance, Epidemiology and End Results (SEER) cancer registries. It was found that for women diagnosed during 1973–82 and irradiated, the cardiac mortality ratio (left versus right tumour laterality) was 1.20 (95% CI 1.04–1.38) less than 10 years afterwards, 1.42 (1.11–1.82) 10–14 years afterwards, and 1.58 (1.29–1.95) after 15 years or more (trend: 2p = 0.03), respectivelly.^26^ For women diagnosed during 1983–92 and irradiated, the cardiac mortality ratio was 1.04 (0.91–1.18) less than 10 years afterwards and 1.27 (0.99–1.63) 10 or more years afterwards. For women diagnosed 1993–2001 and irradiated the cardiac mortality ratio was 0.96 (0.82–1.12) with none yet followed for 10 years. According to the author’s interpretation of the results, since the early 1980s, improvements in radiotherapy planning should have reduced mortality from heart disease in women received radiotherapy.

**FIGURE 1.**
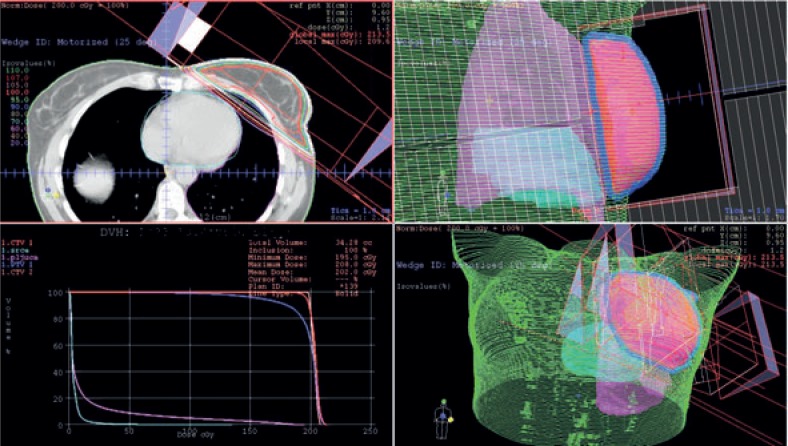
Treatment plan for postoperative irradiation of the left breast in patient with early breast cancer - Three dimensional conformal radiation therapy (3DCRT).

Older radiotherapy techniques (two-dimensional (2D) radiotherapy) and irradiation devices (telecobalt machine), which were used in the past for the treatment of patients with a breast cancer did not allow for as good protection of the heart as it is possible with newer radiotherapy techniques (three-dimensional (3D) conformal radiotherapy) and modern irradiation devices (linear accelerator) (Figure 1). In the future, it is reasonable to expect better outcomes of such studies and less cardiotoxicity.

Despite progress in radiotherapy for the patients with breast cancer, there is still some risk of a cardiac damage due to irradiation. The reason for the damage is the anatomical position of the heart, which lies just below the breast or chest wall, which is irradiated with the therapeutic dose.

### Cardiotoxicity of concomitant radiotherapy and trastuzumab

Preclinical *in vitro* and *in vivo* studies have shown that the cascade of events through the HER2 receptor is involved in tumour radiosensibility^27^, application of trastuzumab concurrently with radiation thus increases the antitumor effect of radiation. There are same clinical evidences in the literature that trastuzumab also radiosensibilizes human healthy tissues and in this way it could increase the toxicity of the treatment.^28^

Currently the most important question remains whether the concomitant therapy with trastuzumab and radiotherapy increases cardiotoxicity of the treatment. In the literature, there are limited data about the safety of concomitant therapy with radiotherapy and trastuzumab. The observation period in the studies was short, the longest reported median observation period after the completion of concomitant treatment with radiotherapy and trastuzumab was 3.7 years.^29^

In the study which included a retrospective series of 218 patients with advanced breast cancer at MD Anderson, a significantly higher rate of cardiovascular events was identified in patients who have completed left breast irradiation, as it was found in patients after irradiation of the right breast (26% *vs.* 7%).^30^ In the multivariate analysis radiation wasn’t shown as an important risk factor for cardiotoxicity, which was observed after the treatment with trastuzumab.

In the monocentric prospective study from the Institute Curie in Paris acceptable skin toxicity and cardiac toxicity was reported after a median observation period of 13 months after the completion of the adjuvant treatment with trastuzumab and concomitant radiotherapy.^11^ In this trial 83% of patients received irradiation not only to the breast or thoracic wall but also to the parasternal lymph nodes. The study involved 106 patients treated between June 2006 and March 2007. Four % of patients developed symptomatic heart failure of whom 2% experienced serious complications related to the heart. Researchers didn’t find significant differences in the effect of the treatment on the skin between the two compered groups of patients (concomitant treatment with trastuzumab and radiotherapy *vs.* only radiotherapy).

NSABP-B31 study did not allow radiotherapy of parasternal lymph nodes. It included 1503 patients and did not show any differences in the incidence of cardiovascular events, regardless of whether the patients were irradiated to the left or right breast/thoracic wall.^31^

Sub-analysis of NCCTG N9831 study has included 1286 patients, of whom in the adjuvant treatment 908 patients received concomitant trastuzumab and radiotherapy and 378 patients received only trastuzumab.^29^ Trastuzumab was administered after the completion of CT with anthracyclines and taxanes. The study shows no significant difference in the frequency of clinically manifest cardiovascular events between the two groups (irradiated *vs*. non-irradiated patients), and there were no significant differences in comparison with the irradiated side.

The Canadian study included 59 patients, of whom 44 were treated with concomitant trastuzumab and radiotherapy.^32^ Median absolute decrease in LVEF after irradiation was 4% between groups (left breast/right breast and with/without parasternal lymph nodes included in the irradiated field), but the study did not show significant differences.

In the HERA study^3^, where there were fewer cardiovascular events than in the NSABP-B31 and NCCTG N9831 trials, the treatment with trastuzumab was started after the completion of CT and radiotherapy, in contrast with the previously mentioned studies, in which trastuzumab was administered concurrently, firstly with CT (except in one study group of NSABP-B31 trial) and then concurrently with radiotherapy.

### Methods for evaluation of cardiotoxicity

The optimal method, duration and frequency of cardiac monitoring for patients receiving trastuzumab combined with radiotherapy have not yet been established. The role of careful history, physical examination, electrocardiograph and chest radiograph is crucial.^33^ Different biomarkers and imaging techniques and their potential role in cardiotoxicity diagnosis have been evaluated in numerous trials. Cardiac troponins and brain natriuretic peptide (BNP) seem to be the most appropriate biomarkers for cardiotoxicity evaluation^34^, while echocardiography and radionuclide ventriculography are imaging techniques that are being most widely used in this setting for the assessment of left ventricular ejection fraction (LVEF). LVEF is the golden standard for monitoring cardiac function in patients receiving cardiotoxic therapy.^35^ The diagnostic importance of other biomarkers such as endothelin-1 or atrial natriuretic peptide^36^–^38^, other imaging techniques such as magnetic resonance imaging^39^ and invasive diagnostic tools such as classical ventriculography with endomyocardial biopsy is limited and are not widely accepted.^37^,^40^

Cardiac troponins I and T are early, highly specific and sensitive markers of myocardial injury.^35^,^37^ Their role in the acute coronary syndrome diagnosis and prognosis is crucial. Elevated levels of troponins can be detected 4–12 hours after the myocardial injury and can persist elevated up to 10 days.^41^ Elevated serum troponins can be detected in variety of other clinical settings such as advanced heart failure, pulmonary embolism, myocarditis, sepsis, arrhythmias, renal failure and also in chemotherapy induced cardiomyopathies. In breast carcinoma patients receiving high doses of anthracyclins, elevated troponins after drug application were related to reduction of LVEF and development of symptomatic heart failure later on.^40^ Those patients were also less likely to recover from heart failure.^42^ On the other hand, the role of troponins in the long-term follow up of these patients is limited because elevated levels do not persist late after the myocardial injury.

BNP is a member of natriuretic hormones family. Their effect is vasodilatative, natriuretic, diuretic and hypotensive. They inhibit renin-angiotensin system and enhance neurohormonal activation in heart failure patients.^37^ BNP is synthesized in the brain and in the ventricles in response to volume overload and consequent ventricular wall distension. After being synthesized, its inactive form proBNP is then cleaved into active BNP and inactive N terminal proBNP (NT-proBNP). Both molecules are being used in heart failure diagnosis, but NT-proBNP is more widely accepted because it is more stable in the serum than active BNP. Also, NT-proBNP levels are higher than BNP levels in heart failure patients comparing to healthy individuals.^43^ NT-proBNP is a sensitive biomarker of both systolic and diastolic heart failure not just as a diagnostic tool but also as a prognostic tool.^37^ Elevated levels can be detected early in the asymptomatic stage of the disease or in patients with the preserved ejection fraction.^34^ Higher levels of NT-proBNP can be detected in patients with low body mass index, women, elderly patients and patients with renal failure or anemia.^37^ Numerous trials tested BNP or NT-proBNP as a diagnostic and prognostic tool for the evaluation of carditoxicity of cancer chemotherapy and radiation therapy. Both hormones proved to be early and sensitive biomarkers of such cardiotoxicity. Patients with elevated NT-proBNP had a higher possibility for asymptomatic LVEF reduction or to develop a symptomatic heart failure later on. Because changes in NT-proBNP are usually earlier than changes in LVEF, its elevated level exposes patients at higher risk. Also in a patient with already developed cardiotoxic effects, the reduction in NT-proBNP carries good prognosis for the improvement in cardiac function. For these reasons NT-proBNP is already widely used as a marker in the evaluation of cardiotoxicity in cancer patients.^35^

**FIGURE 2.**
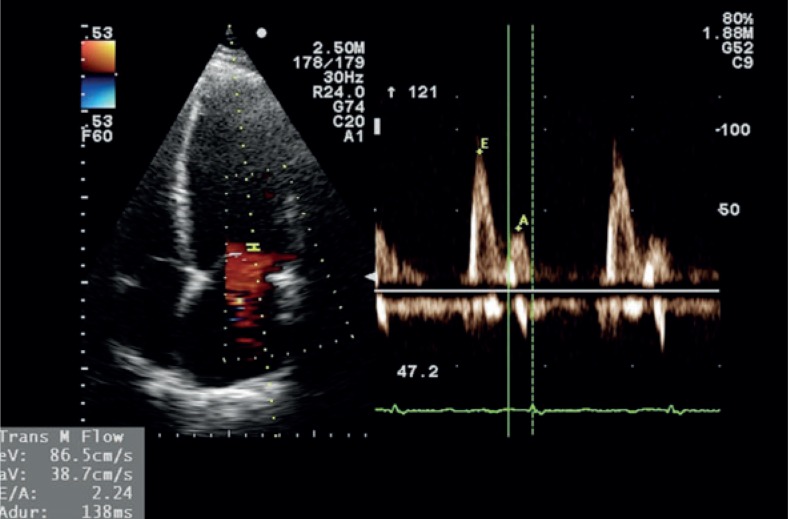
Pulsed wave Doppler measurement of flow velocities through the mitral valve annulus showing normal left ventricular filling pattern. (E = early diastolic flow velocity, A = atrial contraction flow velocity).

**FIGURE 3.**
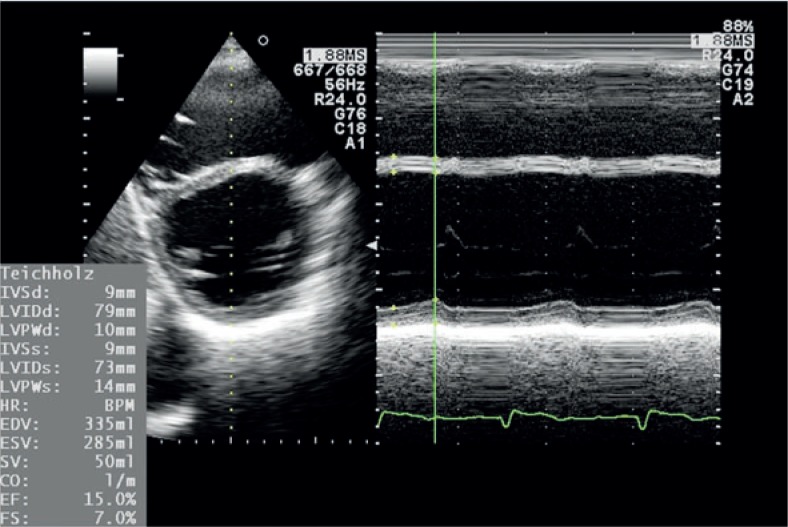
M mode measurement of left ventricular ejection fraction (LVEF) using the Teichholz method from the parasternal short axis view showing extremely enlarged left ventricle with severely reduced LVEF.

**FIGURE 4.**
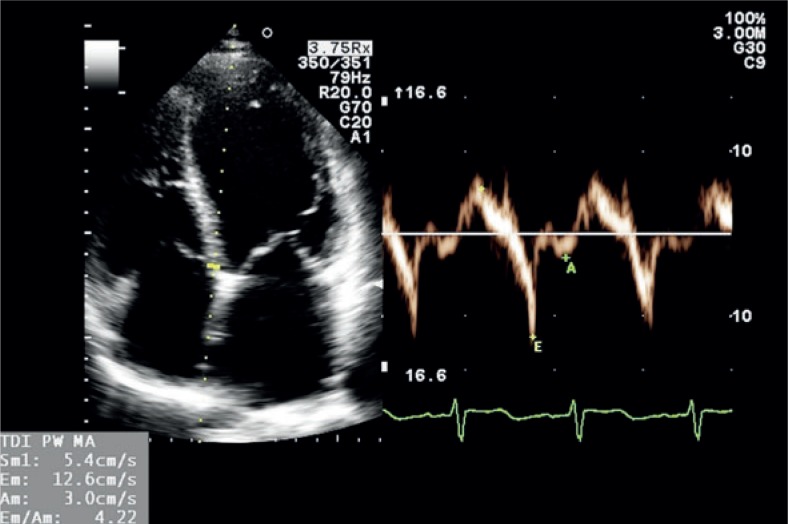
Measurement of tissue Doppler velocities on the mitral annulus from the apical four chamber view showing depressed left ventricular systolic function. (x = systolic velocity, × E = early diastolic velocity, × A = atrial contraction velocity).

Standard transthoracic two-dimensional echocardiography is already a golden standard in the evaluation of carditoxicity in cancer patients.^39^,^44^ It provides useful morphologic and haemodynamic information and not just LVEF alone.^33^,^37^ Measurements of heart chambers and great vessels dimensions, estimation of ventricular systolic and diastolic function, assessment of ventricular wall contraction abnormalities, valvular anatomy and function and diagnosis of pericardial disease are a standard part of echocardiographic exam (Figure 2,3). The limiting factor of echocardiography as a diagnostic tool is its relatively low reproducibility due to high inter- and intraobserver variability.^37^,^40^,^44^ For that reason it is highly recommended for patients that are being followed up for a longer period of time, that serial examinations are performed on the same device by the same echo-cardiographist. Traditionally, LVEF reduction was the only marker of cardiotoxicity in cancer patients. Recently it has been proved that reduction in LVEF is not as sensitive and occurs later than left ventricular diastolic dysfunction.^45^–^47^ Patients with left ventricular diastolic dysfunction before the initiation of the treatment with trastuzumab are at higher risk for developing trastuzumab related cardiotoxicity.^47^ For these reasons a detection of different degrees of left ventricular diastolic dysfunction is of crucial role in early cardiotoxicity diagnosis, especially because some patients never develop ventricular systolic dysfunction (patients with heart failure with preserved ejection fraction). In patients with atrial fibrillation or mitral regurgitation the assessment of ventricular diastolic function can be difficult. In obese patients or in patients after chest irradiation the quality of LVEF measurements can be poor due to suboptimal chest echotranslucency. In these settings tissue Doppler imaging (TDI), a Doppler derived echocardiographic technique measuring myocardial contraction velocities offers additional information regarding left ventricular systolic and diastolic dysfunction (Figure 4). For that reason, many investigators already propose that serial TDI measurements should be a part of a routine echocardiographic examination in cancer patients receiving a cardiotoxic therapy.^39^,^48^,^49^

Another useful diagnostic tool in the evaluation of cardiotoxicity is radionuclide ventriculography. This radionuclear technique uses *in vivo* technetium-99m labelled red blood cells and observes their intracardiac accumulation during different stages of the heart cycle with gamma camera in a standard left anterior oblique view.^47^ It provides highly reproducible and observer independent calculation of LVEF and the assessment of left ventricular diastolic function.^37^,^40^ This method is especially appropriate in conditions mentioned earlier where echocardiography provides less accurate measurements.^44^ That is why some investigators promote radionuclide ventriculography as a golden standard and as a diagnostic method superior to echocardiography in serial evaluation of cardiotoxicity in cancer patients.^38^,^47^ However, comparing to echocardiography radionuclide ventriculography provides solely the information regarding the left ventricular function and no information regarding chamber dimensions, heart valves and pericardium. Its diagnostic role is additionally limited because of the risks related to additional radiation of the patient, especially in the paediatric population.^40^

### Perspectives for the future

Data from the studies published so far indicate that the concomitant therapy with radiation and trastuzumab is likely safety; irradiation should not have a significant additional effect on cardiotoxicity detected after the treatment with trastuzumab. However, so far only data obtained solely some years after the treatment are published. Currently there is no evidence that such therapy is safe even after a long observation period. In the previously mentioned studies biomarker NT-proBNP wasn’t measured, which could possibly earlier than the measurement of LVEF show a significant difference in cardiac toxicity between the two compared groups of patients (concomitant trastuzumab and irradiation to the left/right breast or thoracic wall).

Because we know that trastuzumab in terms of cardiotoxicity in most patients causes only asymptomatic decrease in LVEF^33^ and clinically expressed cardiovascular events do not often occur, there is a need for the trial which would observe heart function after the completion of the treatment with trastuzumab, particularly in combination with radiation, more precisely, as it is only by measuring LVEF. In addition, it is known that 50% of patients with impaired left ventricular function are asymptomatic.^50^ In this group of patients on the basis of clinical symptoms and signs the heart failure or other cardiovascular events cannot be defined, but the treatment success rate of heart failure in this patient group is significantly better.^51^

As yet there are no guidelines for the follow-up of the patients after the treatment with trastuzumab. Perhaps particularly young patients would need a regular follow-up by the cardiologist after the treatment.

Since the new target drugs from the group of HER2 inhibitors (lapatinib, pertuzumab) have been used alone or in combination with trastuzumab, the question of co-toxicity of trastuzumab and radiation is even more important.

### Conclusions

Because the prognosis of patients with HER2-positive early breast cancer, which is considered a more aggressive type of breast cancer, with new types of treatment is improving and their expected lifespan is extending, the determination of the toxicity of treatment has an increasing importance.
